# A survey study on antibiotic prescription practices for acute asthma exacerbations: An European academy of allergy and clinical immunology task force report

**DOI:** 10.1002/clt2.12345

**Published:** 2024-03-18

**Authors:** Anne‐Lotte Redel, Wojciech Feleszko, Alessandra Arcolaci, Francesca Cefaloni, Marina Atanaskovic‐Markovic, Gert‐Jan Braunstahl, Cristina Boccabella, Matteo Bonini, Aspasia Karavelia, Eefje Louwers, Norbert Mülleneisen, Liam O'Mahony, Laura Pini, Anna Rapiejko, Esmeralda Shehu, Milena Sokolowska, Eva Untersmayr, Gerdien Tramper‐Stranders, EAACI Task Force on Conscious and Rational use of Antibiotics in Allergic Diseases

**Affiliations:** ^1^ Department of Pulmonology Franciscus Gasthuis & Vlietland Rotterdam The Netherlands; ^2^ Department of Pulmonology Erasmus Medical Center Rotterdam The Netherlands; ^3^ Department of Pediatric Allergy and Pneumonology Medical University of Warsaw Warsaw Poland; ^4^ Immunology Unit University of Verona and General Hospital Borgo Roma Hospital Verona Italy; ^5^ Università Cattolica del Sacro Cuore Rome Italy; ^6^ Faculty of Medicine University of Belgrade University Children's Hospital Belgrade Serbia; ^7^ Faculty of Medicine and Surgery Respiratory Medicine Catholic University of the Sacred Heart Milan Italy; ^8^ Department of Cardiovascular and Thoracic Sciences Università Cattolica del Sacro Cuore Fondazione Policlinico Universitario A. Gemelli ‐ IRCCS Rome Italy; ^9^ National Heart and Lung Institute Royal Brompton Hospital & Imperial College London London UK; ^10^ Department of Ear‐Nose‐Throat Surgery General Hospital of Nafplio Nafplio Greece; ^11^ General Practice Gezondheidscentrum Berkel en Rodenrijs Berkel en Rodenrijs The Netherlands; ^12^ Asthma‐Allergiezentrum Leverkusen Leverkusen Germany; ^13^ Department of Medicine School of Microbiology APC Microbiome Ireland National University of Ireland Cork Ireland; ^14^ Department of Clinical and Experimental Sciences Respiratory Medicine Unit University of Brescia ASST Spedali Civili di Brescia Brescia Italy; ^15^ Internal Medicine Department Durres Regional Hospital Durres Albania; ^16^ Swiss Institute of Allergy and Asthma Research (SIAF) University of Zurich Zürich Switzerland; ^17^ Institute of Pathophysiology and Allergy Research Center of Pathophysiology Infectiology and Immunology Medical University of Vienna Vienna Austria; ^18^ Department of Pediatrics Franciscus Gasthuis & Vlietland Rotterdam The Netherlands

**Keywords:** antibiotics, asthma, exacerbation, survey

## Abstract

**Introduction:**

Guidelines recommend treating asthma exacerbations (AAEs) with bronchodilators combined with inhaled and/or systemic corticosteroids. Indications for antibiotic prescriptions for AAEs are usually not incorporated although the literature shows antibiotics are frequently prescribed.

**Aim:**

To investigate the antibiotic prescription rates in AAEs and explore the possible determining factors of those practices.

**Methods:**

A digital survey was created to determine the antibiotic prescription rates in AAEs and the influencing factors for the prescription practices. The survey was distributed among European academy of allergy and clinical immunology (EAACI) members by mass emailing and through regional/national societies in the Netherlands, Italy, Greece, and Poland. Furthermore, we retrieved local antibiotic prescription rates.

**Results:**

In total, 252 participants completed the survey. Respondents stated that there is a lack of guidelines to prescribe antibiotics in AAEs. The median antibiotic prescription rate in this study was 19% [IQR: 0%–40%] and was significantly different between 4 professions: paediatrics 0% [IQR: 0%–37%], pulmonologists 25% [IQR: 10%–50%], general practitioners 25% [IQR: 0%–50%], and allergologists 17% [IQR: 0%–33%]) (*p* = 0.046). Additional diagnostic tests were performed in 71.4% of patients before prescription and the most common antibiotic classes prescribed were macrolides (46.0%) and penicillin (42.9%). Important clinical factors for health care providers to prescribe antibiotics were colorised/purulent sputum, abnormal lung sounds during auscultation, fever, and presence of comorbidities.

**Conclusion:**

In 19% of patients with AAEs, antibiotics were prescribed in various classes with a broad range among different subspecialities. This study stresses the urgency to compose evidence‐based guidelines to aim for more rational antibiotic prescriptions for AAE.

## 1 INTRODUCTION

Prescribing antibiotics is influenced by the knowledge of healthcare providers, local guidelines, and the availability of the medication. To reduce the antibiotic prescription rate in patients with an acute asthma exacerbation (AAE) and determine specific criteria for prescription, an overview of the prescription rates practices and beliefs is needed. Asthma is one of the most common respiratory diseases and affects 1%–18% of the children and adults worldwide.^1^ In 2019, this airway disease caused 455,000 deaths globally.^2^ This heterogeneous airway disease is characterised by the combination of chronic airway inflammation and a variable expiratory airflow limitation. Asthma patients present with respiratory symptoms, such as wheeze, shortness of breath, chest tightness, and cough that vary in time and intensity.^1^

An AAE is an acute worsening of the asthma symptoms and can be induced by respiratory infections, allergic stimuli, air pollutants and other environmental factors.^3^, ^4^, ^5^ Asthma exacerbations can significantly impact the quality of life, resulting in hospitalisations and absenteeism from school or work.^6^ The Global Initiative for Asthma (GINA) guidelines recommend bronchodilators combined with inhaled and/or systemic corticosteroids for the treatment of AAEs, without prescribing antibiotics unless there is a strong suspicion of bacterial infection.^1^ However, antibiotics are often prescribed despite the lack of evidence, and local practices vary largely.^7^, ^8^ The overuse of antibiotics should be reduced due to increasing antimicrobial resistance, negative effects on the commensal microbiota, and rising health costs worldwide.^9^ To address these concerns and improve antibiotic prescription practices, there is an urgent need for criteria/guidelines to structure this practice in AAEs.

The use of antibiotics in acute Chronic Obstructive Pulmonary Disease (COPD) exacerbations also poses a concern. In Italy, a survey was distributed among general practitioners and pulmonologists to evaluate the antibiotic prescription practices in patients with COPD exacerbations. For general practitioners and pulmonologists, different clinical signs were important for prescribing antibiotics. The clinical factor that was most important for general practitioners was purulent sputum, while for pulmonologists, the most important factors were body temperature and increased C‐reactive protein (CRP).^10^

The European Academy of Allergology and Clinical Immunology (EAACI) task force ‘Conscious and rational use of antibiotics in allergic diseases’ aims to diminish unnecessary antimicrobial agent use. The task force previously published knowledge gaps and future research directions with respect to antibiotic use related to allergic conditions.^11^ In order to develop guidelines to reduce the antibiotic prescription rate in patients with an AAE, an overview of the prescription practices, beliefs and current criteria for prescription is needed. We hypothesised that prescribing practices range widely and vary among countries and different subspecialities due to a lack of guidelines. This EAACI survey study aimed to investigate the antibiotic prescription rates for AAEs and explore possible determining factors of those practices for different medical specialities and countries. Data collected will aid in future guideline development by the task force.

## 2 METHODS

### 2.1 Study population

Data were collected using an online questionnaire among different physicians working in the field of respiratory and/or allergy medicine worldwide from June until September 2022. The e‐mail with the online survey was opened by 4703 members of the European academy of allergy and clinical immunology (EAACI) by mass emailing. The questionnaire was also translated into Greek, Italian, and Polish and was distributed by task force members locally and/or nationally.

### 2.2 Questionnaires and procedures

The EAACI task force ‘Conscious and rational use of antibiotics in allergic diseases’ created a survey about the antibiotic prescription rates in patients (paediatric or adult) diagnosed with AAE. The survey was formulated using the literature and is added as Supporting Information S1: (Appendix 1). The survey consisted of questions concerning prescriber demographics, such as age and participant's professions, as well as their actual number of prescriptions, attitudes, and practices towards antibiotic prescriptions in AAEs. To calculate the antibiotic prescription rate for the participants, the survey included questions about how many AAEs the participants had diagnosed in the last week and month and how many of these patients antibiotics were prescribed.

The online survey was initiated by obtaining informed consent from the participants. The participants were allowed to withdraw from participation by discontinuing the survey before completion. Only the completed surveys were included for analysis and the data were analysed anonymously. The survey was created in the GCP‐proof electronic data capture system Castor Electronic Data Capture (Castor EDC).

In addition, participants were invited to provide their contact details if they consented to being approached for further study on the objective numbers of AAE and antibiotic prescriptions within their organisation anonymously. The participants were invited to share the absolute numbers of AAEs per month for the designated period of April until October 2022. They were asked to specify how many of the patients were treated with antibiotics and which antibiotic class was prescribed.

### 2.3 Statistics

Descriptive analyses were performed to evaluate the demographics and baseline characteristics. Based on the distribution of the variables, the numerical variables were presented as mean with standard deviation or as median with interquartile range. Categorical variables were presented as frequencies or proportions.

The antibiotic prescription rate was defined as the proportion or percentage of antibiotics prescribed in the total number of patients diagnosed with an AAE within a specific time window (week or month). Participants who did not diagnose an AAE were excluded from this analysis. The antibiotic treatment in AAE was evaluated as a dichotomous variable. If health care providers prescribed two or more antibiotic treatments for one AAE, then it was calculated as one prescription. The antibiotic prescription rates were compared per profession with the Kruskal–Wallis test. Several participants shared objective numbers of AAEs and antibiotic prescriptions in their practices. These proportions were calculated for the overall period from April to October 2022. Statistical analyses were performed using IBM SPSS Statistics v.28.0.

## 3 RESULTS

### 3.1 International survey and proportion of antibiotic prescriptions

The international survey distributed by the EAACI was completed by 112 health care providers from 49 different countries. The respondents worked in Europe (74.1%), Asia (11.6%), Oceania (5.4%), North‐America (4.5%), Africa (2.7%), and South‐America (1.8%). The median age was 48.0 years [IQR: 36.0–59.0] and their median years of practice was 18.0 years [IQR: 9.3–28.8]. The most represented professions among the 112 respondents were allergologists (62.5%), paediatricians (20.5%) and pulmonologists (10.7%) (see Table 1).

**TABLE 1:** Demographic characteristics of all the respondents.

	International survey	National surveys				
	EAACI (*n* = 112)	Poland (*n* = 93)	The Netherlands (*n* = 22)	Greece (*n* = 13)	Italy (*n* = 12)	Total group (*n* = 252)
Demographic data respondents						
Age	48.0	49.0	43.0	41.0	33.0	46.5
	[36.0–59.0]	[32.4–57.5]	[39.6–49.0]	[31.5–50.5]	[29.3–35.0]	[36.0–57.0]
Years of practice	18.0	19.0	11.5	13.0	4.5	17
	[9.3–28.8]	[4.0–29.0]	[7.8–21.8]	[5.5–18.5]	[3.3–10.3]	[7.0–25.0]
Profession						
Allergologist	70 (62.5%)	19 (20.4%)	0	0	0	89 (35.3%)
Paediatrician	23 (20.5%)	28 (30.1%)	0	0	2 (16.7%)	53 (21.0%)
Pulmonologist	12 (10.7%)	19 (20.4%)	0	2 (15.4%)	9 (75%)	42 (16.7%)
General practitioner	2 (1.8%)	20 (21.5%)	22 (100%)	2 (15.4%)	0	46 (18.3%)
ENT (‐surgery)	0	1 (1.1%)	0	6 (46.2%)	0	7 (2.8%)
Internal medicine	1 (0.9%)	4 (4.3%)	0	0	0	5 (2.0%)
Other	4 (3.6%)	2 (2.2%)	0	3 (23.1%)	1 (8.3%)	10 (4.0%)
Acute asthma exacerbations data						
Number of respondents with AAE treatment	105	90	17	12	10	234
Number of AAEs	1163	613	36	100	51	1963
Number of AB prescriptions	358	144	18	19	6	545
Median proportion AB prescriptions	0.18	0.20	0.33	0.17	0	0.19
	[0–0.33]	[0–0.46]	[0–0.50]	[0.02–0.88]	[0–0.23]	[0–0.40]

*Note*: Age and years of practice are described as median and IQR. The frequencies of participants per profession are presented as absolute numbers (and percentage) per survey. The AAE data is based on respondents who diagnosed AAE in the last month. The antibiotic prescription rates are presented as median and IQR. The last column shows the total of all separate surveys.

**FIGURE 1:**
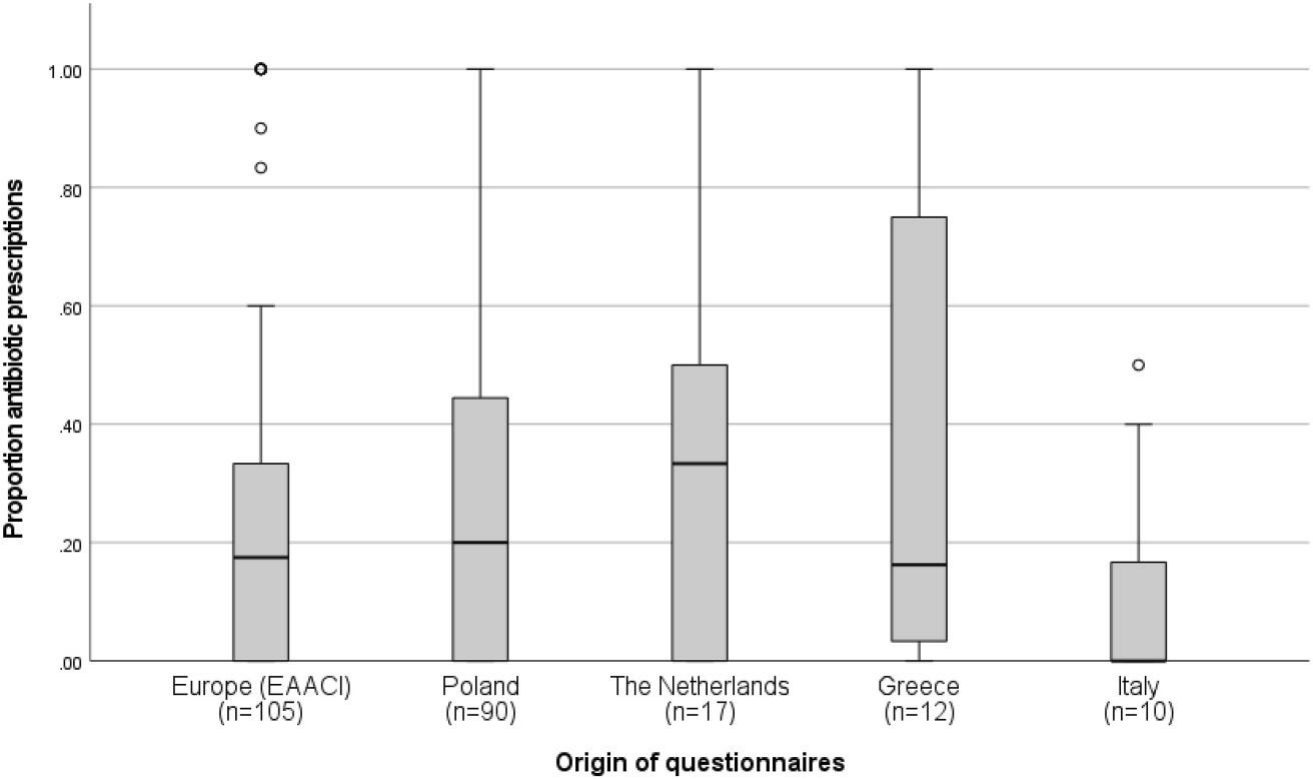
Proportion antibiotic prescriptions in acute asthma exacerbations for the international survey (EAACI) and the different national surveys, presented as boxplots with a median and IQR. The sample size of the AAE is presented. Only participants who diagnosed an AAE are included. AAE, acute asthma exacerbation.

Based on the international survey, 105 of the 112 participants diagnosed patients with an AAE during the month before the completing the survey. The calculated proportion of antibiotic prescriptions for these patients was 0.18 [IQR: 0–0.33] (see Figure 1 and Table 1). Most of the participants of the international survey (72.3%) stated that the number of AAEs varied per season and among these participants, 49% indicated that the current season was a quiet season.

**FIGURE 2:**
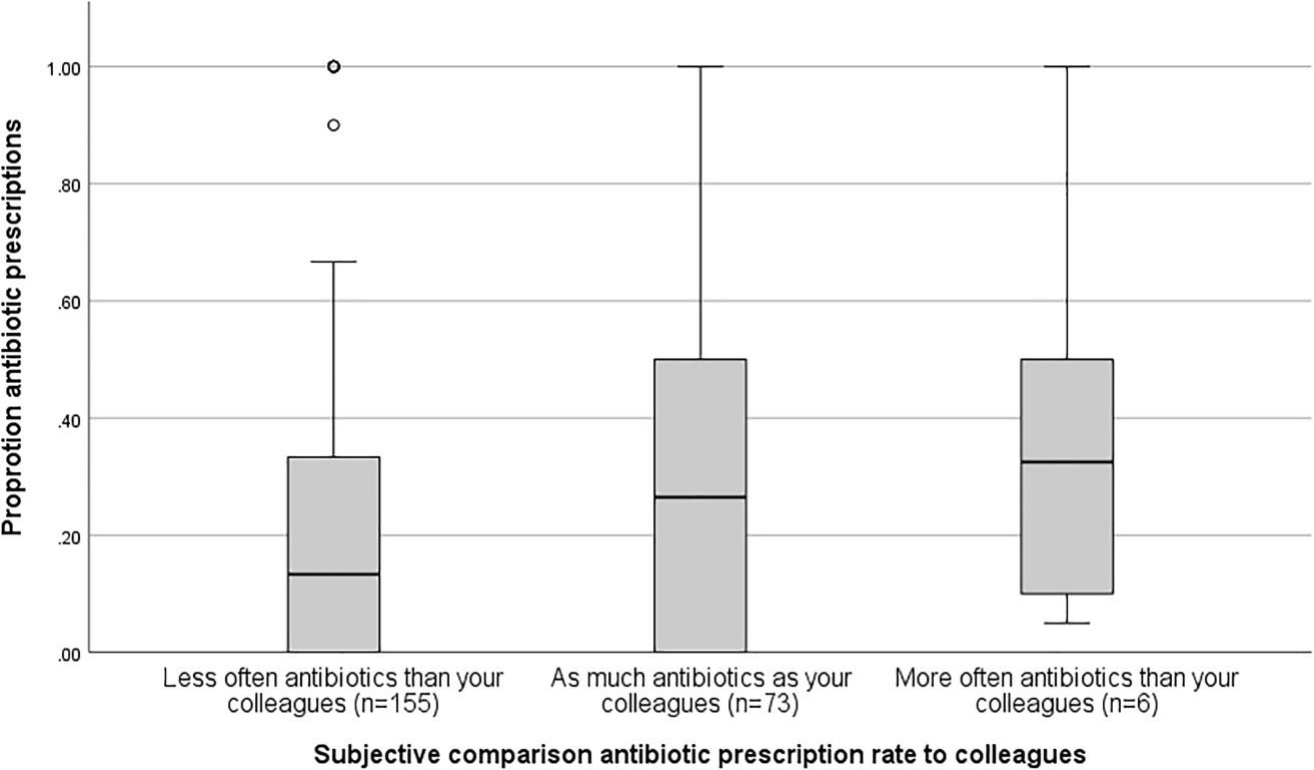
Participants' estimations about the antibiotic prescription practices compared with their colleagues. Data based on the total of the international and national surveys.

In total, 80.6% of all the respondents (both national and international surveys) stated that there is a lack of guidelines or criteria to prescribe antibiotics in AAE. Next, we asked participants whether they assume they prescribe antibiotics almost often compared to their colleagues. The medians of the participants who thought they prescribed more (median 0.33; IQR [0.09–0.63]) or as much antibiotics as their colleagues (median 0.26; IQR [0.0–0.50]) were higher than the overall median proportion of 0.19 [IQR: 0–0.40]. Figure 2 shows, how participants compare their prescription rates to their colleagues.

### 3.2 Local/national surveys and proportion of antibiotic prescriptions

We received completed surveys from Poland (*n* = 93), The Netherlands (*n* = 22), Greece (*n* = 13) and Italy (*n* = 12). Table 1 shows the median age, the years of practice, and the distribution of professions for the different surveys. The proportion antibiotic prescriptions in AAE varied for the different nations (see Figure 1 and Table 1). After merging the data of the international and national questionnaires, the overall proportion of antibiotic treatments in AAEs was 19% [IQR: 0%–40%] (*n* = 234).

### 3.3 Considerations to prescribe antibiotics and antibiotic classes

The most important factors to consider when prescribing antibiotics were colorized/purulent sputum (82.5%), the reduction of lung sounds or the presence of crackles (61.9%), increased body temperature (>38.5°C) (52.8%), and the presence of comorbidities (51.2%). Before prescribing, 71.4% performed additional diagnostic tests, mainly chest X‐ray, blood CRP or leucocytes, and sputum cultures. Participants used different cut‐offs for CRP and leucocyte levels to support antimicrobial prescriptions in AAE patients. Independent of the clinical recovery, 12.7% of the participants performed additional diagnostic tests after the AAE treatment. However, 48.8% of the participants performed diagnostic tests after antibiotic prescription only if a patient had not fully recovered.

**FIGURE 3:**
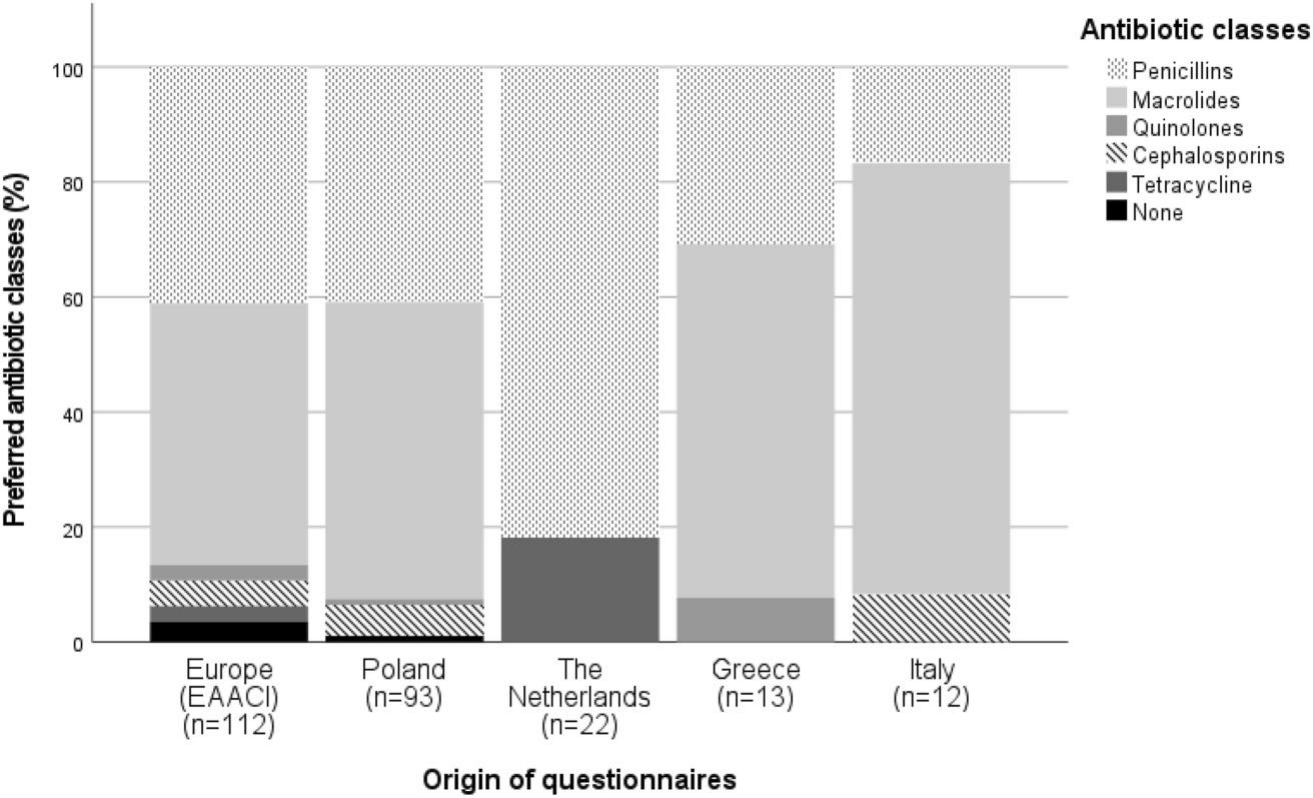
The most common prescribed antibiotics in AAE based on the first choice of prescriber, divided by international survey (presented as EAACI) and the different national surveys. The percentages were calculated as the percentage of the total completed questionnaires per type of questionnaire. None = participants who did not prescribe antibiotics in AAEs. AAE, acute asthma exacerbation.

Macrolide (46.0%), penicillin (42.9%), and cephalosporin antibiotics (4.4%) were the most commonly prescribed classes (Figure 3). The median duration of the prescription was 7.0 days [IQR: 6.0–7.0] for penicillin and 7.0 days [IQR: 5.0–7.0] for macrolides. Figure 3 also demonstrates similar outcomes in preference for antibiotic classes across various national surveys. However, the Dutch general practitioners did not prescribe macrolides in contrast to the other participants.

Based on the international survey, 18.8% of the participants reported that antibiotics were available without a prescription from a health care provider, primarily from Europe and Asia. For the national surveys 1.5% of health care providers (working in Italy) indicated that antibiotics are available over the counter.

### 3.4 Antibiotic prescriptions differentiated per profession

**FIGURE 4:**
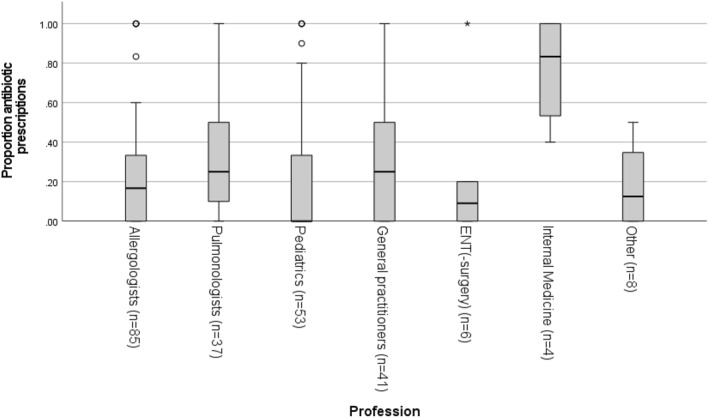
Proportion antibiotic prescriptions in AAEs per profession. Proportions are illustrated as boxplots, including the median and IQR. Only health care providers who diagnosed AAE were included. AAE, acute asthma exacerbation.

After merging the results of all the surveys, the following four main professions were analysed separately: allergologists, pulmonologists, paediatricians and general practitioners. Figure 4 presents the proportion of antibiotic prescriptions for AAEs per profession, which was significantly different between these professions (*p* = 0.046). It was lower for paediatricians (0, IQR: [0–0.37]) compared to pulmonologists (0.25, IQR: [0.10–0.50]), allergologists (0.17, IQR: [0–0.33]), and general practitioners (0.25, IQR: [0–0.50]).

### 3.5 Objective antibiotic prescription rates in AAE

Seven organisations provided data on the actual numbers of AAE and antibiotic prescriptions during April‐October 2022. The proportions varied between 0.09 and 1 with an overall median of 0.22 (IQR: 0–0.50) (see Table 2).

**TABLE 2:** Actual proportion of antibiotic prescriptions in AAE from April to October 2022 per organisation.

Country	Organisation	Population	Total AAEs	Proportion antibiotic prescriptions
Albania	Regional Hospital	Children (age from 14–18 years)	5	0.20
		Adults	59	0.15
Germany	Asthma and Allergy Centre	Adults	20	0.55
Italy	University Medical Centre	Children and adults	15	0.67
Italy	Respiratory Medicine Unit	Adults	5	1.00
Serbia	University Children's Hospital	Children (age from 6–16 years)	32	0.09
The Netherlands	Asthma and Allergy Centre Hospital	Adults	477	0.24
The Netherlands	General Practitioner Practice	Adults	21	0.43

## 4 DISCUSSION

This is the first report of multinational perceptual and actual antibiotic practices for asthma exacerbations. By using the EAACI network and regional/national societies, we were able to collect 252 responses from 49 different countries, with a focus on Europe.

In both the survey study and more detailed actual practice study, the percentage of antibiotic prescriptions varied widely and were on average 19%. Interestingly, we found a significant difference between the four biggest professions in our dataset. Paediatricians prescribed fewer antibiotics in patients with AAEs compared with pulmonologists, general practitioners, and allergologists.

The majority of participants indicated that there is a lack of criteria or guidelines for prescribing antibiotics in AAEs. The international consensus of paediatric asthma does not describe the role of antimicrobial treatment in children diagnosed with AAEs.^12^ Furthermore, the GINA guidelines refer to considering prescribing antibiotics to patients in presence with fever, purulent sputum, or radiographic evidence of pneumonia, but otherwise do not provide guidance.^1^ Antimicrobial stewardship and guidelines for AAE are therefore highly needed.

Previous studies report that prescription rates are dependent on health care setting, region and patient profile. A retrospective study performed in the United States describes an antibiotics prescription rate of 22% with a significant lower antibiotics prescription rate in specific regions of the United states, in urban hospitals, and in African American patients.^13^ In our study, health care providers who believe they prescribe fewer antibiotics or prescribe at a similar rate to colleagues actually underestimate their own prescription frequency in AAEs. Health care providers are aware of the increasing antimicrobial resistance problem, but they often believe that it is caused by others' practices.^14^

Macrolide and penicillin antibiotics were the most frequently prescribed antibiotics for AAE. The prescription of fluoroquinolones and cephalosporins for AAE is noteworthy. Both of those antibiotic classes are associated with emerging resistance and should be used with caution if there are appropriate alternatives. Macrolide antibiotics are probably also being prescribed because of their immune modulatory properties, but pneumococcal macrolide resistance is rising, especially in the Southern and Eastern regions of Europe.^15^ Because of rising resistance, macrolides are not advised as empiric treatment for pneumonia anymore. This is reflected in the results of the Dutch general practitioners in this study.^16^

The wide range of antibiotic treatments for AAEs could be influenced by diagnostic uncertainty. Most of the health care providers in this study performed additional diagnostic tests before prescribing antibiotics, but the majority of those tests, such as chest X‐ray and CRP, did not differentiate between viral and bacterial infection. Diagnostic uncertainty is described as a risk factor for a higher antibiotic prescription rate.^17^

Other factors that may influence antibiotic practices in AAE are time pressure for the health care provider and financial considerations. Antibiotics could protect further complications and increase costs, and might be considered in patients in lower resource settings in which adequate follow‐up might be difficult.^18^ These factors were not investigated in this study.

Antimicrobial stewardship programs are associated with a reduced proportion of antibiotic prescriptions.^19^ There are conflicting results in the literature about the effect of education on prescription practices. A randomised controlled trial was performed in the primary care in Switzerland, where the intervention group received an audit and feedback on their antibiotic practices in general. This intervention did not result in a lower antibiotics prescription rate compared to the control group.^20^ However, a review summarised studies in the primary and secondary care investigating antibiotic prescriptions for different indications. Antibiotic treatments could be reduced by interventions such as education, prescription audits, training in communication skills, and prescription recommendations from experts.^21^

Next to antimicrobial stewardship, further research into clinical algorithms and/or biomarkers is needed to identify those AAE patients who would benefit from antibiotics. The previous Azalea trial did not show benefit from macrolide treatment in all AAE.^22^ Currently, the EXCLUSIEF study (NCT05304039) is addressing this question by phenotyping AAE in detail and relating it to the treatment response.

There are several limitations to this study. Due to the wide distribution of this survey, the number of respondents for individual countries was too small to compare the prescription rates between different countries. The calculated prescription rates can be influenced by population bias because of the distribution among EAACI members. Moreover, the low participation rate and the respondent bias might be present by selection of those physicians already interested in antimicrobial stewardship, leading to a distorted outcome compared to a general sample of physicians. This is probably reflected by the data from Italy, where respondents described low prescription rates while the rates in the objective number study were much higher (0%–23% vs. 67%). Higher prescription rates are also described in the literature, both among children and adults.^7^ Larger datasets are needed to compare prescription practices and investigate other factors influencing prescription rates such as costs and intrinsic beliefs in order to measure the effects of antimicrobial stewardship. In further research, it would be recommended to collect objective numbers of patients with an AAE, including the ages of the patients. With this data, it would be possible to analyse the effect of age on antibiotic prescription practices in primary and secondary care and the different underlying factors between age groups. Furthermore, the differences in professions could be influenced by the different countries in which the participants work (see Table 1). The majority of the paediatricians in our study work in Poland and the general practitioners work in the Netherlands and Poland.

## 5 CONCLUSION

About 19% of patients with an asthma exacerbation are being prescribed antibiotics, of various classes, with a broad variety among different medical subspecialities. There is a lack of clear recommendations on antibiotics for AAE in guidelines. This study stresses the urgency to compose evidence‐based guidelines to aim for more rational antibiotic prescriptions for AAE. Till then, we would advocate for every respiratory/allergy physician to regularly review their local antibiotic prescription rate for AAE to create awareness of potentially unnecessary prescriptions.

## AUTHOR CONTRIBUTION

Creating the survey: Anne‐Lotte Redel, Wojciech Feleszko, Alessandra Arcolaci, Marina Atanaskovic‐Markovic, Gert‐Jan Braunstahl, Cristina Boccabella, Matteo Bonini, Aspasia Karavelia, Liam O'Mahony, Milena Sokolowska, Eva Untersmayr, and Gerdien Tramper‐Stranders. Data collection: Anne‐Lotte Redel, Wojciech Feleszko, Francesca Cefaloni, Marina Atanaskovic‐Markovic, Cristina Boccabella, Matteo Bonini, Aspasia Karavelia, Eefje Louwers, Norbert Mülleneisen, Laura Pini, Anna Rapiejko, Esmeralda Shehu, and Gerdien Tramper‐Stranders. Data analysis and draft manuscript: Anne‐Lotte Redel and Gerdien Tramper‐Stranders.

## CONFLICT OF INTEREST STATEMENT

None of the authors of this survey study report any conflicts of interest related to this study.

## Supporting information

Supporting Information S1

## Data Availability

The data that support the findings of this study are available from the corresponding author upon reasonable request.
