# Supplementary material for: A survey study on antibiotic prescription practices for acute asthma exacerbations: An European academy of allergy and clinical immunology task force report
Source: Clin Transl Allergy. 2024 Mar 18;14(3):e12345. doi: 10.1002/clt2.12345 (PMC10946284; doi:10.1002/clt2.12345)
Supplement: Supplementary file 1 — Supporting Information S1 [file CLT2-14-e12345-s001.docx]

**Appendix 1: Questionnaire (English version)**

**Introduction**

Dear EAACI members,

The EAACI task force on **‘Conscious and rational use of antibiotics in allergic diseases’** is investigating antibiotic prescription trends for asthma exacerbations. As the asthma exacerbation season is intensifying after lifting of pandemic restrictions, this period will be perfect to register antibiotic prescription practices. We therefore kindly invite EAACI members (specifically pulmonologists, allergologists, paediatricians and general practitioners) to fill in this short survey (approximately 10 minutes). It would be of great help to our task force to understand factors associated with antibiotic prescriptions for asthma exacerbations to aid in writing a position paper/guideline for a rational prescribing policy.

Also, we are looking for practitioners willing to help in providing more specific anonymous data about hospital/practice prescription data (to be pooled), e.g. generated by their business intelligence unit. If interested, leave your email address in the survey (optional).

Thank you in advance!

**Informed consent**

The collected data of this survey will be stored in an electronically secured software system, Castor EDC. By completing this survey, you give your consent in providing (confidential) information, which will be analysed anonymously. Participating in this survey is voluntary and you may withdraw your participation by closing the questionnaire before submission. Data will be archived in the software system for 15 years and will be destroyed afterwards. **Do you grant consent to use your (anonymous) answers on this survey.**

**Yes**

**No**

Please write down today’s date: ____________________________

**Background information**

1. What is your age?

____________________________years

1. Since when do you practice as a medical specialist? Please write down the year that you started as a medical specialist.

____________________________

1. What country are you working in? ^[[1]](#footnote-1)^

____________________________

1. What is your current profession?
   1. Allergologist
   2. Pulmonologist
   3. Paediatrician
   4. Emergency physician
   5. General practitioner
   6. Other: ____________________________
2. Where do you practice?
   1. Peripheral/secondary hospital
   2. Academic/tertiary hospital
   3. General practice/primary care
   4. Private practice
   5. Other: ____________________________

**Acute asthma exacerbations and prescription rate**

1. How many patients did you evaluate in the **last week** and diagnosed with an acute asthma exacerbation? (Please note an absolute number)

____________________________patients in the last week

1. How many patients diagnosed with an acute asthma exacerbation received antibiotics in the **last week**? (Please note an absolute number)

____________________________patients in the last week

1. How many patients did you evaluate in the **last month** and diagnosed with an acute asthma exacerbation? (Please note an absolute number)

____________________________patients in the last month

1. How many of your acute asthma exacerbation patients received antibiotics in the **last month**? (Please note an absolute number)

____________________________patients in the last month

1. Do the number of antibiotic prescriptions change at different times of the year? ^[[2]](#footnote-2)^
   1. Yes
   2. No

10.1 Is it now a busy, an average or a quiet time of year for antibiotic prescriptions? ^[[3]](#footnote-3)^

a. Busy season

b. Average season

c. Quiet season

1. If you compare your rate of antibiotic prescriptions for acute asthma exacerbations with the prescriptions of your colleagues,
   1. You think you prescribe more often antibiotics than your colleagues
   2. You think you prescribe as much antibiotics as you colleagues
   3. You think you prescribe less often antibiotics than your colleagues
2. Which class of antibiotics do you mostly prescribe for patients with an acute asthma exacerbation?
   1. Penicillins (e.g. amoxicillin, amoxicillin/clavulanic acid)
   2. Macrolides (e.g. azithromycin, erythromycin, clarithromycin)
   3. Quinolones (e.g. Ciprofloxacin, levofloxacin, moxifloxacin)
   4. Cephalosporins (e.g. Cefaclor, cefuroxime, ceftibuten)
   5. Tetracycline (e.g. doxycycline)
   6. Other, ____________________________
3. What is the average duration of the antibiotic prescription (in days) that you prescribe for patients with an acute asthma exacerbation?

____________________________ days

**Guidelines**

1. Robust and reliable literature evidence exist to support the use of antibiotics for acute asthma exacerbations. Do you agree or disagree?
   1. Agree
   2. Disagree
2. Do you have **local** guidelines concerning the treatment of acute asthma exacerbation in your hospital or health care institution?
   1. Yes
   2. No
3. Do you have a **national** guidelines recommending antibiotic treatment for acute asthma exacerbations?
   1. Yes
   2. No
4. Do the local and/or national guidelines detail the eligibility criteria for prescribing antibiotics in patients with an acute asthma exacerbation?
   1. No
   2. Yes, in the local guidelines
   3. Yes, in the national guidelines
   4. Yes, in the local and national guidelines

**Patient history and physical examination**

1. In presence of which factors do you prescribe antibiotics (next to oral corticosteroids)? Select the 5 most important factors:
   1. Temperature > 38 ᵒC
   2. Temperature > 38,5 ᵒC
   3. Runny nose
   4. Pharyngitis
   5. Sat O2 < 95%
   6. Sat O2 < 90%
   7. Respiratory rate > 30/min (in adults)
   8. Respiratory rate > 24 /min (in adults)
   9. Cough
   10. Reduction in lungs sounds or presence of crackles
   11. Difficulty in speaking
   12. Diffuse wheezing
   13. Purulent or colorized sputum
   14. Absence of symptom improvement after inhalation of beta-2 agonists (salbutamol)
   15. Excessive participation of thoracic muscles in respiration (evident jugular veins, paradoxical pulse, subcutaneous crepitating – rare physical symptoms during severe asthma or status asthmatic).
   16. Comorbidities

18.1. In the previous question, you selected ‘comorbidities’ as a factor to prescribe antibiotics next to corticosteroids. What are the three most common comorbidities in your practise to prescribe antibiotics for an acute asthma exacerbation? ^[[4]](#footnote-4)^

____________________________

**Complementary information**

1. Do you perform additional diagnostic tests before prescribing antibiotics for patients with an acute asthma exacerbation?
   1. No
   2. Yes
2. If you perform additional diagnostic tests before prescribing antibiotics, which test or tests do you perform? You are allowed to select more than one test. ^[[5]](#footnote-5)^
3. C-reactive protein
4. Procalcitonin
5. Peripheral blood leucocytes
6. Chest X-ray
7. Sputum culture
8. Other: ____________________________

20.1. Which cut-off value (and unit of measurements) for C-reactive protein do you use to prescribe antibiotics? ^[[6]](#footnote-6)^

____________________________

20.2. Which cut-off value (and unit of measurements) for procalcitonin do you use to prescribe antibiotics? ^[[7]](#footnote-7)^

____________________________

20.3. Which cut-off value (and unit of measurements) for leucocytes do you use to prescribe antibiotics? ^[[8]](#footnote-8)^

____________________________

20.4. Which characteristics of the chest X-ray do you use to consider antibiotics prescription in patients with an acute asthma exacerbation? ^[[9]](#footnote-9)^

____________________________

20.5. Do you initiate antibiotics after receiving the results of the sputum culture?

1. Yes
2. No, I start the antibiotics while waiting for the results of the sputum culture
3. Do you perform additional diagnostic tests after the antibiotic treatment for patients diagnosed with acute asthma exacerbation?
   1. No
   2. Yes, always after treatment
   3. Yes, only if the patient did not fully recover after treatment

21.1. Which diagnostic test do you perform after antibiotic treatment? ^[[10]](#footnote-10)^

____________________________

**Availability of antibiotics**

1. In your country, is it possible for patients to receive antibiotics over the counter (without a prescription)?
   1. Yes
   2. No

**Contact**

We want to evaluate the prescription rate of antibiotics in patients with an acute asthma exacerbation. Therefore we would like to collect the antibiotics prescription numbers from different health care settings and different countries. Are you or your business intelligence/ICT unit able to extract an overview of the number antibiotic prescriptions for acute asthma exacerbations?

Please write down your e-mail address and we will contact you for further details.

E-mail address: ____________________________

1. This question is a multiple choice question in castor including 197 different countries. [↑](#footnote-ref-1)
2. Question 10.1 will only appears if the participant answers yes in question 10 [↑](#footnote-ref-2)
3. This question appearrs when the participant selects ‘yes’ at question 10. [↑](#footnote-ref-3)
4. Question 18.1 will only appears if the participant selected ‘p’ as one of the options in question 18. [↑](#footnote-ref-4)
5. Questions 20.1 – 20.5 will only appear depending on the answer to question 20. Only the options that are selected by the participant, will appear as the following question. [↑](#footnote-ref-5)
6. This question appears if answer ‘a’ is selected in question 20 [↑](#footnote-ref-6)
7. This question appears if answer ‘b’ is selected in question 20. [↑](#footnote-ref-7)
8. This question appears if answer ‘c’ is selected in question 20. [↑](#footnote-ref-8)
9. This question appears if answer ‘d’ is selected in question 20. [↑](#footnote-ref-9)
10. This question will only appears if the participant answers b or c for question 21. [↑](#footnote-ref-10)
